# Silicon-Controlled Rectifier Embedded Diode for 7 nm FinFET Process Electrostatic Discharge Protection

**DOI:** 10.3390/nano12101743

**Published:** 2022-05-19

**Authors:** Xinyu Zhu, Shurong Dong, Fangjun Yu, Feifan Deng, Kalya Shubhakar, Kin Leong Pey, Jikui Luo

**Affiliations:** 1 Hangzhou Global Scientific and Technological Innovation Center, Zhejiang University, Hangzhou 310027, China; 11931028@zju.edu.cn (X.Z.); 21960364@zju.edu.cn (F.Y.); 22060270@zju.edu.cn (F.D.); jackluo@zju.edu.cn (J.L.); 2 Engineering Product Development, Singapore University of Technology and Design, Singapore 138682, Singapore; shub0002@ntu.edu.sg (K.S.); peykinleong@sutd.edu.sg (K.L.P.)

**Keywords:** silicon-controlled rectifier, diode, FinFET, ESD

## Abstract

A new silicon-controlled rectifier embedded diode (SCR-D) for 7 nm bulk FinFET process electrostatic discharge (ESD) protection applications is proposed. The transmission line pulse (TLP) results show that the proposed device has a low turn-on voltage of 1.77 V. Compared with conventional SCR and diode string, the proposed SCR-D has an additional conduction path constituting by two additional inherent diodes, which results in a 1.8-to-2.2-times current surge capability as compared with the simple diode string and conventional SCR with the same size. The results show that the proposed device meets the 7 nm FinFET process ESD design window and has already been applied in actual circuits.

## 1. Introduction

Electrostatic discharge (ESD)-induced damage is one of the main failures of integrated circuits. In the 7 nm FinFET process used in the state-of-the-art integrated circuits, the gate oxide of FinFET is extremely fragile under an electrostatic discharge (ESD) impact due to its reduced thickness and low reliability of high-k dielectric [1,2,3], and the ESD protection will degrade gradually after encountering non-fatal ESD strikes [4,5]. Some techniques for ESD modeling and simulation have been utilized in the FinFET process to help analyze the characteristics of ESD protection under ESD strike [6,7,8,9]. The ESD protection diodes are considered to be promising in advanced technology as an ESD protection device [6,7,8]. The diode-string silicon-controlled rectifier (DSSCR) with high robustness is also considered as the ESD protection device for previous technology nodes [10,11,12,13,14,15], but it is no longer suitable for the 7 nm technology due to its high leakage and large snapback for latch-up. ESD design for the FinFET process is still a great challenge. A high-robustness ESD-protection device with a sufficiently low trigger voltage (Vt) and high failure current (It2) is not yet available. In this letter, we propose a new silicon-controlled rectifier-embedded diode (SCR-D) based on the 7 nm FinFET process. The characteristics of this protection with different key designs are fabricated and analyzed.

## 2. Design and Analysis

**Figure 1:**
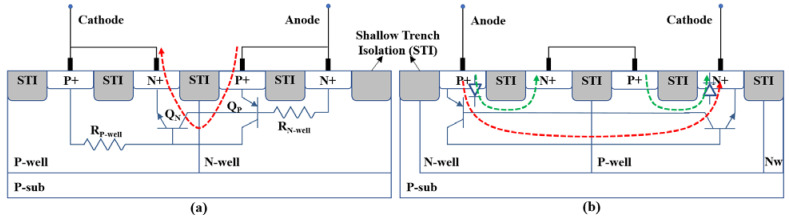
Cross-sectional views of SCR structures: (**a**) conventional SCR device;(**b**) SCR-D proposed in this work. Color lines indicate the current flow when it is turned on.

Figure 1a shows the cross-sectional view of a conventional SCR composing two bipolar transistors with two parasitic well resistors. When the SCR is turned on, the positive feedback path formed by the two transistors enables a large current flow between the cathode and anode to bypass the ESD current with a very small size; however, the turn-on time of the SCR is very long in the order of 20 ns, and the trigger voltage is too high to fit in the design windows of MOS technologies. In the modified structure SCR-D shown in Figure 1b, we introduce diodes to both the cathode and anode to reduce the trigger voltage. In contrast to Figure 1a, the P-well and N-well of the SCR-D are swapped, as is the cathode and anode, although the P+ and N+ junctions connected to the terminals and the fabrication process remain unchanged. This new arrangement results in two additional PN junctions and two different bipolar transistors. On the anode side, the PNP transistor is constituted by P+, N-well, and P-well, whereas the NPN transistor on the cathode side is constituted by N+, P-well, and N-well. This structure effectively increases the distance between cathode and anode, thus resulting in a larger holding voltage and a smaller snapback voltage.

**Figure 2:**
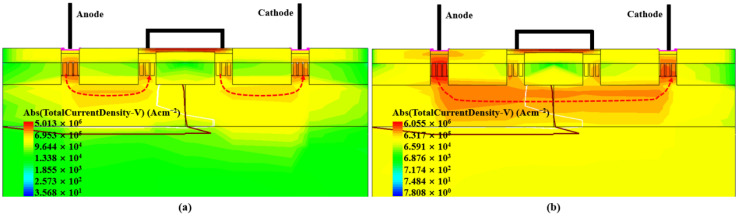
TCAD simulated current distribution of SCR-D at different stages: (**a**) low ESD current; (**b**) large ESD current.

Two-dimensional technology computer-aided design (TCAD) simulation was carried out to illustrate the operation of the devices. Figure 2 shows the current distribution of the SCR-D for two different situations. Figure 2a shows the low-level current conduction case. The major current-conduction path is via the anode diode (P+/N-well junction), to the metal wire connecting N-well and P-well and then the cathode diode (P-well/N+ junction). The two-diode string can release the initial ESD current. As the diode current in the anode increases to a certain level and triggers the avalanche breakdown of the reversely biased PN junction between the N-well and P-well (see Figure 2b), the SCR will be turned on. A significant amount of current flows from the anode to cathode via the N-well and P-well, and the current level of the conduction becomes high.

## 3. Result and Discussion

### 3.1. TLP Results

**Figure 3:**
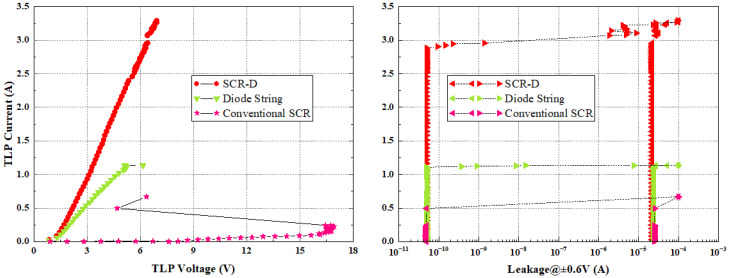
TLP I-V characteristics and leakage measured from diode string, conventional SCR, and SCR-D proposed in this work.

The TLP I-V characteristics of the diode string, conventional SCR and SCR-D are shown in Figure 3. It is noted that the conventional SCR is broken down almost immediately when it is turned on at 16 V. For the forward-biased two-diode string, although the turn-on voltage is 1.4 V, which can meet the 7 nm FinFET application requirement (1.1 V to 5 V), the failure current is very low (~1.2 A), which is not particularly suitable for the application. Increasing the failure current requires larger sizes of the diodes, which will result in a larger leakage current. For the new SCR-D structure, the trigger voltage is lowered to 1.77 V. The failure current (It2) of SCR-D increases to about 2.25 A, and the leakage current is below nA level because of the much smaller size of the diodes. Table 1 compares the characteristics of these three ESD protection devices. To have a fair comparison, we define the failure current per device area as the robustness figure-of-merit (FOM), as shown in the last column Table 1. For the same device area, SCR-D provides 80% more current-sinking capability than the two-diode string. The sinking current of SCR-D is about 2.2 times of that of the conventional SCR with the same device area.

### 3.2. Optimization Device Sturcture

**Figure 4:**
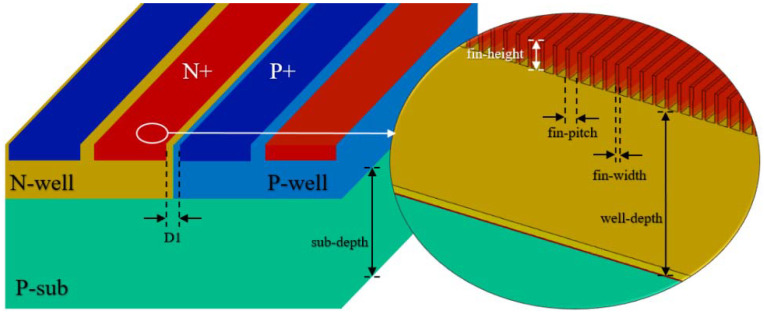
SCR-D parameters in TCAD simulation; enlarged view of Fin structure.

Having verified the operation mechanism of the SCR-D in FinFET process with TCAD simulation, we will next simulate the effects of device parameters on the ESD protection characteristics of the SCR-D device. Different from the planar process, we need to consider the three-dimensional fin structure in FinFET process in the simulation. Figure 4 shows some parameters for SCR-D and parameters of the fin structure for TCAD simulation. We focus on the impact of the variation of key parameters on the device performance, such as the spacing between the N+ and P+ regions, which is represented as D1 in Figure 4. Table 2 shows the intrinsic parameters in FinFET process, which are fixed irrelevant to integrated circuit layout and are used in the TCAD simulation.

**Figure 5:**
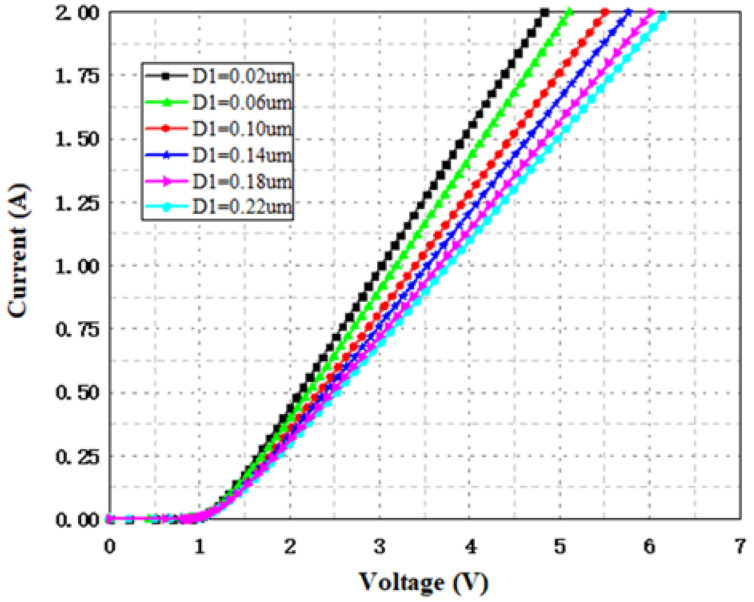
I-V curves for SCR-D in TCAD simulation with different D1 values.

The lower trigger voltage and on-state resistance indicate that SCR-D has the combined advantages of the conventional SCR and diode string. When an ESD pulse comes, the trigger diode string conducts first, which takes part of the trigger voltage, and then the SCR path is turned on, providing a low-resistance current discharge through not only through the surface of silicon but also the bulk. Figure 5 simulates the I-V curve of SCR-D with different D1 values. The simulation results are summarized in Table 3, which show that a smaller D1 value is required to reduce the trigger voltage and on-state resistance.

## 4. Conclusions

A new silicon-controlled rectifier embedded diode (SCR-D) is proposed for 7 nm FinFET process by swapping the N-well and P-well in the conventional SCR process. The new SCR-D introduces two additional diodes that provide an additional low-current conduction path to reduce the trigger voltage to about 1.77 V and enhance the maximum conducting current by about 1.8-to-2.2 times, respectively, as compared with the diode string and conventional SCR with the same device size. These parameters meet the design window specifications as derived from the 7 nm FinFET process. The proposed SCR-D does not need any additional process steps and can be applied in an actual circuit.

## Figures and Tables

**Table 1:** Comparison of the key parameters of three ESD protection devices.

Device	Trigger Voltage (V)	It2 (A)	Robustness (mA/μm^2^)
SCR	16.9	0.10	2.70
2-diode string	1.40	1.20	3.30
SCR-D	1.77	2.25	5.96

**Table 2:** The intrinsic parameters in FinFET process used in simulation.

Size Name	Value (μm)	Doping Region	Value (cm^3^)
sub-depth	10.000	P-sub	1 × 10^15^
well-depth	0.450	P-well	1.9 × 10^18^
fin-height	0.070	N-well	1.9 × 10^18^
fin-width	0.008	P+	1.2 × 10^21^
fin-pitch	0.030	N+	1.2 × 10^21^

**Table 3:** Comparison of the key parameters of SCR-D with different D1 values.

**D1 (μm)**	0.02	0.06	0.10	0.14	0.18	0.22
**Trigger Voltage (V)**	1.324	1.351	1.385	1.404	1.419	1.436
**On-State Resistance (Ω)**	1.81	1.92	2.11	2.24	2.37	2.45

## Data Availability

Not applicable.

## References

[1] Wong H., Iwai H. (2015). On the scaling of subnanometer EOT gate dielectrics for ultimate nano CMOS technology. Microelectron. Eng..

[2] Wong H., Zhou J., Zhang J., Jin H., Kakushima K., Iwai H. (2014). The interfaces of lanthanum oxide-based subnanometer EOT gate dielectrics. Nanoscale Res. Lett..

[3] Wong H. (2012). Nano-CMOS Gate Dielectric Engineering.

[4] Wong H., Dong S., Chen Z. On the ESD Protection and Non-Fatal ESD Strike on Nano CMOS Devices. Proceedings of the IEEE 31st International Conference on Microelectronics (MIEL).

[5] Wu J., Rosenbaum E. (2004). Gate oxide reliability under ESD-like pulse stress. IEEE Trans. Electron Devices.

[6] Li Y., Miao M., Gauthier R. ESD Protection Design Overview in Advanced SOI and Bulk FinFET Technologies. Proceedings of the IEEE Custom Integrated Circuits Conference (CICC).

[7] Linten D., Hellings G., Chen S.-H., Groeseneken G. ESD in FinFET technologies: Past learning and emerging challenges. Proceedings of the IEEE International Reliability Physics Symposium (IRPS).

[8] Chen S.-H. ESD Challenges in Advanced FinFET and GAA Nanowire CMOS Technologies: Designing Diode Based ESD Protection in Advanced State of the Art Technologies. Proceedings of the IEEE Custom Integrated Circuits Conference (CICC).

[9] Li Y., Wang Y., Wang Y. ESD Diode Devices Simulation and Analysis in a FinFET Technology. Proceedings of the International EOS/ESD Symposium on Design and System (IEDS).

[10] Song S., Du F., Hou F., Song W., Liu Z., Liu J. A New dual directional SCR with high holding voltage for High Voltage ESD protection. Proceedings of the IEEE International Conference on Electron Devices and Solid-State Circuits (EDSSC).

[11] Huang X., Liu Z., Liu F., Liu J., Song W. (2017). High holding voltage SCRs with segmented layout for high-robust ESD protection. Electron. Lett..

[12] Lin L., Gauthier R., Loiseau A., Lu X. Design optimization of a breakdown silicon controlled rectifier (BDSCR) for cell phone antenna switch pin electrostatic discharge (ESD) protection. Proceedings of the Electrical Overstress/Electrostatic Discharge Symposium Proceedings.

[13] Liang H., Xu Q., Zhu L., Gu X., Sun G., Lin F., Zhang S., Xiao K., Yu Z. (2019). Design of a Gate Diode Triggered SCR for Dual-Direction High-Voltage ESD Protection. IEEE Electron Device Lett..

[14] Lai D.-W., Tseng W.-J., De Raad G., Smedes T. DNW-controllable triggered voltage of the integrated diode triggered SCR (IDT-SCR) ESD protection device. Proceedings of the 39th Electrical Overstress/Electrostatic Discharge Symposium (EOS/ESD).

[15] Du F., Song W., Hou F., Liu J., Liu Z., Liou J.J., Xiong X., Li Q., Liu Y. (2020). Augmented DTSCR With Fast Turn-On Speed for Nanoscale ESD Protection Applications. IEEE Trans. Electron Devices.

